# Nutrition and Lifestyle Behaviours for the Prevention and Management of Multiple Sclerosis

**DOI:** 10.3390/nu17233685

**Published:** 2025-11-25

**Authors:** Tyler J. Titcomb, Yasmine Probst

**Affiliations:** 1 Department of Dietetics and Nutrition, University of Kansas Medical Center, Kansas City, KS 66160, USA; 2 Department of Neurology, University of Kansas Medical Center, Kansas City, KS 66160, USA; 3 School of Medical, Indigenous and Health Sciences, University of Wollongong, Wollongong, NSW 2500, Australia

Multiple sclerosis (MS) is a chronic, immune-mediated, neurodegenerative disease that affects over 2.9 million people worldwide. In recent years, lifestyle behaviours have garnered significant interest in the community of MS researchers and patients, due to their recognized role in improving wellness and managing symptoms. However, given the inconsistencies observed within the evidence, many questions remain, including those regarding the role of lifestyle behaviours in the prevention of MS, the impact of lifestyle behaviours on disease progression, the mechanisms by which lifestyle behaviours can improve disease outcomes, and the role that lifestyle behaviours play in the clinical management of MS. This Special Issue, titled “*Nutrition and Lifestyle Behaviours for the Prevention and Management of Multiple Sclerosis*”, provides new insights into these questions through six original research articles and a narrative review.

Two original research articles explored the associations between nutrition-related biomarkers and MS outcomes. Stojkovic et al. observed that, among participants with progressive MS, erythrocyte fatty acid profiles were correlated with measures of disability in a cross-sectional study. Notably, total saturated fatty acid levels were correlated with expanded disability status scale (EDSS) values. Furthermore, this study observed that palmitic acid (16:0), total *n*-3 poly-unsaturated fatty acids (PUFAs), and several individual *n*-3 PUFAs were associated with the MS severity scale (MSSS), and that supplementation with *n*-3 fatty acids may favourably modify erythrocyte fatty acid profiles [1]. The study by Wicks et al. utilized proton nuclear magnetic resonance spectroscopy to compare the metabolite profiles of people with MS to those of healthy controls, cross-sectionally. The researchers computed composite indices from the identified metabolites and observed that the Inflammatory Vulnerability Index, the Metabolic Malnutrition Index, and the Metabolic Vulnerability Index differed between the healthy control and MS groups. Furthermore, among the sample with MS, the Metabolic and Inflammatory Vulnerability Indexes were associated with EDSS, timed ambulation, lower grey matter and deep grey matter volumes, and greater lateral ventricle volumes [2].

The other studies addressed elements of lifestyle management for MS. These studies investigated nutrition knowledge, food literacy, and inaccuracies in dietary reporting. Additionally, other considerations, including body composition, disordered eating, self-esteem and body dissatisfaction, as well as the utilization of referrals to registered dietitian nutritionists for MS care, were explored. For example, Rienmann-Lorenz et al. developed and validated a nutrition knowledge and food literacy screening tool with 148 people living with MS; this tool demonstrated satisfactory discriminatory power in the food literacy questions. Strong correlation outcomes between the MS-specific food literacy tool and a general food literacy tool were demonstrated, although only weak correlations were evident for the nutrition knowledge tool, this potentially being due to the low nutrition knowledge among the sample [3]. The authors acknowledged a need for educational interventions, which also form a key part of dietetic consultations. However, these consultations are often challenged in actual practice by referral rates to dietitians, as outlined by Wills et al.: of the 60 MS healthcare professionals included in the survey, more than half reported that their patients with MS inquired about their diet during their consultations, with MS healthcare professionals highlighting a topic that warrants further exploration. In addition, almost three in four (73.9%) MS healthcare professionals reported having referred their patients to a dietitian for general healthy eating advice, while over two in three (67.4%) reported having referred them to a dietitian for weight management support [4]. These findings highlight the need for MS-specialized, registered dietitians being involved in MS care teams.

Health professional teams working in MS care are required to consider a range of factors for managing the health and wellbeing of people living with MS, from the direct management of symptoms through to the social interactions, societal implications and other indirect impacts on the person. The concept of the MS-specific qualifications of health professionals entails an evidently advanced understanding and focus on the condition, with advice and support strategies oriented toward a better-tailored and disease-specific focus. For disease-specific directions that health professionals may engage in for MS, consideration should be given to how these vary from other chronic conditions. Within a dietetic consultation, for example, a dietary assessment may be conducted by a dietitian to gain insights into the patterns of eating that a person displays. In a healthy population, these assessments are completed with an understanding that misreporting is likely for demographic groups, though for MS, the added challenge that verbal learning and memory can have on a recall of food and beverage intake needs to be considered alongside the misreporting. Silvera et al. explored this in their study of 28 people living with MS, comparing an online 24 h dietary recall to the gold-standard energy expenditure assessment via the doubly labelled water test to address the accuracy of reporting by exploring differences in total energy expenditure and reported energy intake. The study identified that verbal learning and memory, as well as light physical activity, impact self-reported energy intake, although only the verbal learning outcomes could explain the percentage difference in self-reported energy intake [5]. These findings, in addition to the review on body composition presented by Kidwell-Chandler et al., suggest that a greater focus on body composition is necessary in randomized controlled trials for MS and can help to create a framework for clinical practice and future research in MS [6].

For dietitian and psychologist healthcare professionals, this framework may also consider additional factors such as body dissatisfaction, self-esteem and neuroticism, which were each related to signs of disordered eating in a study by Kiropoulos et al. [7], an area that has limited evidence in MS. The research published in this Special Issue included 275 participants living with MS who completed a range of validated questionnaires. The controlled mediation analysis demonstrated that neuroticism and disordered eating are explained by body dissatisfaction and self-esteem, suggesting new areas for screening within MS healthcare teams. This screening process suggests areas for further research whereby interdisciplinary and multidisciplinary team involvement in areas such as eating disorders and, potentially, malnutrition can be trialled in practice settings to advance existing models of care for MS.
